# Synergistic effects of silybin and curcumin on virulence and carbapenemase genes expression in multidrug resistant *Klebsiella oxytoca*

**DOI:** 10.1186/s13104-022-06172-3

**Published:** 2022-10-22

**Authors:** Farah H. Omer, Noor S. K. Al-Khafaji, Farah Tareq Al-Alaq, Hussein O. M. Al-Dahmoshi, Mojtaba Memariani, Morteza Saki

**Affiliations:** 1 Department of Clinical Laboratory Science, College of Pharmacy, University of Mosul, Mosul, Iraq; 2 Department of Biology, College of Science, University of Babylon, Hilla, Iraq; 3 Skin Research Center, Shahid Beheshti University of Medical Sciences, Tehran, Iran; 4 Department of Microbiology, Faculty of Medicine, Ahvaz Jundishapur University of Medical Sciences, Ahvaz, Iran; 5 Infectious Ophthalmologic Research Center, Imam Khomeini Hospital Clinical Research Development Unit, Ahvaz Jundishapur University of Medical Sciences, Ahvaz, Iran

**Keywords:** Silybin, Curcumin, Antimicrobial effects, Virulence genes, *Klebsiella oxytoca*

## Abstract

**Objective:**

Silybin and curcumin have potential antimicrobial effects. This study aimed to evaluate the synergistic antimicrobial effects of silybin and curcumin on virulence and carbapenemase genes expression among multidrug-resistant (MDR) *Klebsiella oxytoca*.

**Results:**

A total of 70 MDR *K. oxytoca* (carrying *bla*_IMP_ and *bla*_OXA-48-like_ genes) were included. The antibiotic susceptibility and biofilm production of isolates were determined. The silybin and curcumin at concentrations 10–500 mg/mL alone and in combination were exposed to bacterial isolates in Mueller Hinton broth medium for 24 h. The expression of *bla*_IMP_, *bla*_OXA-48-like_, *mrkA*, *pilQ*, *matB* and *fimA* genes was evaluated using quantitative real-time polymerase chain reaction (qRT-PCR).

The mean minimum inhibitory concentration (MIC) of curcumin and silybin were 250 mg/mL and 500 mg/mL, respectively. The anti-virulent effect of 100 mg/mL of silybin and curcumin was shown by significant reduction in the expression of *fimA* (2.1-fold, *P* < 0.0001) and *mrkA* (2.1 fold, *P* < 0.0001) genes. Moreover, these compounds significantly decreased the expression of *bla*_IMP1_ (3.2-fold, *P* < 0.0001) gene. Notably, there was no significant effect on *pilQ*, *matB* and *bla*_OXA-48-like_ genes. The results showed that silybin and curcumin can be candidate as natural way for control the MDR virulent strains of *K. oxytoca*.

**Supplementary Information:**

The online version contains supplementary material available at 10.1186/s13104-022-06172-3.

## Introduction

Multidrug-resistant (MDR) *Enterobacteriaceae* members are causative agents of fatal nosocomial infections which have narrow or none therapeutic choices [1–3]. The evolution of carbapenemase-producing (CP) strains has limited last-line treatment resorts. These strains may develop the resistance through various mechanisms such as production of carbapenemase enzymes [2, 3]. Some of these enzymes include imipenemases (IMPs), OXA-type beta-lactamase, and New Delhi metallo-β-lactamase 1 (NDM-1) [4].

MDR *Klebsiella oxytoca* with resistance to carbapenems has been reported from various areas which encode carbapenemase genes [5, 6]. *K. oxytoca* strains are mostly opportunistic pathogens among immunocompromised patients [7]. Biofilm formation is another strategy of *K. oxytoca* for cell attachment and colonization. In *K. oxytoca*, *fimA, mrkA, pilQ* and *matB* play substantial role in bacterial attachment and colonization [3, 8]. FimA, MrkA and MatB are the major protein subunits of type 1 (*fimBEAICDFGH* operon), type 3 (*mrkABCDF* operon) and Mat fimbriae, respectively which recognized in uropathogenic *Escherichia coli* and can mediate adhesion and biofilm formation [9–11]. PilQ is one of the structural subunits of type IV pili (T4P) that is involved in several processes such as motility, biofilm formation, and DNA uptake [12].

One strategy to combat the infections caused by the virulent and drug-resistant bacteria is control of expression of thier genes. Hence, seeking to alternative approaches such as herbal medicine compounds contribute to efficient eradication of infections. Silybin is a multiple applicable herbal medicine compound and has antimicrobial properties [13]. Furthermore, curcumin is another herbal compound with vast properties [14–16]. The antimicrobial effects of curcumin have been determined previously [15, 16]. Also, combination anticancer therapies using curcumin-silybin has been demonstrated before [17].

Until today, there has not been any previous study regarding effect of curcumin and silybin on the virulence and carbapenemase genes expression. This study aimed to assess the effects of silybin and curcumin on virulence and carbapenemase genes expression among MDR *K. oxytoca*.

## Main text

### Materials and methods

#### Ethics

This study was approved by the University of Mosul, Mosul, Iraq according to the Declaration of Helsinki. No human and animal data or sample were included in this study.

#### Clinical isolates

The flow chart of the employed procedures in the present study is shown in Additional file 1: Fig. S1 [18] to the readers at one glance. Herein, 70 MDR *K. oxytoca* isolates that collected from stool samples during 2012–2019 were included. The isolation and identification of *K. oxytoca* were performed using standard bacteriology tests including MacConkey agar (Merck, Germany), triple sugar iron agar, methyl red/Voges-Proskauer, and citrate and urea utilization as mentioned in Table 1 [19]. The antibiotic susceptibility pattern was determined using Kirby-Bauer method according to the Clinical and Laboratory Standards Institute (CLSI) 2017 [20]. The antibiotic discs included cefepime (30 μg), cefotaxime (30 μg), ceftazidime (30 μg), ciprofloxacin (5 μg), tetracycline (30 μg), amikacin (30 μg), piperacillin-tazobactam (100/10 μg), gentamicin (10 μg), imipenem (10 μg), meropenem (10 μg), and trimethoprim-sulfamethoxazole (1.25/23.75 μg) (Bioanalyse, Ankara, Turkey). *Escherichia coli* ATCC^®^ 25922^TM^ was used as quality control strain. Isolates that were resistant against three antibiotics in different classes were considered MDR [21]. All isolates carried the *bla*_IMP_, *bla*_OXA-48-like_, *mrkA*, *pilQ*, *matB* and *fimA* genes.

**Table 1:** Biochemical test results for *Klebsiella oxytoca* isolates

Test	Results
Growth on MacConkey agar	Pink mucoid colonies
Gram staining	Pink-red coccobacilli
Growth on triple sugar iron agar	Acid/acid; Gas positive; H2S negative
Methyl red	Negative
Voges-Proskauer	Positive
Citrate utilization	Positive
Urease	Positive
Lysine decarboxylase	Positive
Arginine dehydrolase	Negative
Ornithine decarboxylase	Negative

#### Curcumin and silybin antibacterial effects

Curcumin and silybin compounds were purchased from Sigma Aldrich, USA. Curcumin (1,7-bis-(4-hydroxy-3-methoxyphenyl)-hepta-1,6-diene-3,5-dione) is a yellow–orange lipophilic compound that dissolves in acetone, dimethylsulfoxide, and ethanol but is insoluble in water, acidic, and neutral solutions. This compound is an integral component of turmeric (*Curcuma longa*) (up to ~ 5%) [22]. This aromatic ginger family (*Zingiberaceae*) plant is native to southwestern and southern Asia [22]. It consists of two aromatic rings symmetrically substituted in *ortho* position with methoxy and phenolic OH groups. These 2 rings are joined to a seven-membered hydrocarbon chain with an enone portion and 1,3-diketone group [22]. For thousands of years, milk thistle (*Silybum marianum*) has been used as an herbal remedy for treating a variety of diseases [23]. Among the major components of *S*. *marianum* fruit extract (silymarin) is a flavanolignan called silybin, which is the most active principal constituent [23]. Molecular formula of silybin is C_25_H_22_O_10_ and its molecular weight is 482.441. It is also called silybine, flavobin, and silymarin I. In general, it is a highly functionalized small molecule with alternate carbo- and hetero-cycles [23]. There are two main units in the silybin structure: the first unit is based on a flavononol group in flavonoids, called taxifolin, and the second is conyferil alcohol, a phenyllpropanoid unit. An oxeran ring joins the two units together to form one structure [23]. Various concentrations (10–500 mg/mL) of both compounds were prepared and their minimum inhibitory concentration (MIC) and minimum bactericidal concentration (MBC) values were determined according to previously described method [24]. The Mueller Hinton broth (MHB) medium (Merk, Germany) was employed. A bacterial suspension equal to 0.5 MacFarland turbidity was prepared and added to each dilution and incubated at 37 °C for 24 h. The MIC of a concentration was defined following observation of no growth and the MBC was defined for each dilution without growth of 100 µL of suspension onto the MHA medium [24]. *Escherichia coli* ATCC^®^ 25922™ was used as quality control strain.

#### Biofilm formation

The biofilm formation was conducted using microtiter plate assay. A bacterial suspension was cultured into each well of 96-well plate containing trypticase soy broth (TSB) medium (Merck, Germany) and incubated for 24 h. The plates were washed and dried. The well attachments were fixed using methanol. The crystal violet was added and left for 15 min and washed again. Using ethanol, the bacterial attachments were made soluble and the opacity was measured using ELISA reader at OD490. The biofilm formation was defined compared to the control wells. There were four categories of isolates: non-biofilm producers (OD_T_ < ODc), weak-biofilm producers (ODc < OD_T_ < 2 × ODc), moderate-biofilm producers (2×ODc < OD_T_ < 4 × ODc), and strong-biofilm producers (4 × ODc < OD_T_) [25].

#### RNA extraction and real-time polymerase chain reaction

The total RNA (Qiagen GmbH, Hilden, Germany) was extracted from 1 × 10^6^ cells suspension of isolates and cDNA (Takara, Japan) was synthesized according to the manufacturers’ instructions. The quantitative real-time polymerase chain reaction (qRT-PCR) was performed using specific primers represented in Table 2 and using CFX96 Touch Real-Time PCR Detection System (Bio-Rad, USA). The *gyrA* gene was considered as the reference of expression analysis (Table 2) [26]. The *K. oxytoca* ATCC^®^ 43165™ was used as control strain.

**Table 2:** The sequence of primers used in this study

Primer	Sequence: 5′–3′	Product size (bp)	Annealing (°C)	References
*bla*_IMP_	F: GGGTGGGGCGTTGTTCCTA R: TCTATTCCGCCCGTGCTGTC	198	62	[5]
*bla*_OXA-48_	F: TGTTTTTGGTGGCATCGAT R: GTAAMRATGCTTGGTTCGC	177	45	[5]
*mrkA*	F:CTGGCCGGCGCTACTGCTAAG R:CACCCGGGATGATTTTGTTGG	172	61	[5]
*fimA*	F: GCACCGCGATTGACAGC R: CGAAGGTTGCGCCATCCAG	132	61	[5]
*matB*	F:GTACTGGGCGGCAACCTTAG R:GTGCCGCTGATGATGGAGAA	98	61	[5]
*pilQ*	F: TCCGCCAGGCTCCACTTC R: GCTCGCGGGCATCTGAC	194	61	[5]
*gyrA*	F: CGCGTACTATACGCCATG AACGTA R: ACCGTTGATCACTTCGGTCAGG	–	57	[26]

#### Statistical analysis

Chi Square and analysis of variance (ANOVA) tests were used with 95% confidence intervals to analyze the data using Statistical Package for the Social Sciences (SPSS) version 22.0 (IBM Corporation, Armonk, NY, USA). A *P*-value less than 0.05 was considered significant [8].

### Results

#### Antibiotic resistance

As shown in Table 3 and Additional file 2: Fig. S2, all isolates were resistant to ceftazidime, cefotaxime, imipenem, tetracycline and trimethoprim-sulfamethoxazole and considered MDR. Moreover, 98.6%, 90.0%, 90.0%, 81.4%, 80.0% and 80.0% of them were resistant to meropenem, piperacillin-tazobactam, amikacin, ciprofloxacin, cefepime, and gentamicin, respectively. The highest rate of susceptibility was to cefepime (20.0%).

**Table 3:** Antibiotic susceptibility of *Klebsiella oxytoca* isolates

Antibiotics	Resistant N (%)	Intermediate N (%)	Susceptible N (%)
Ceftazidime	70 (100.0)	0 (0.0)	0 (0.0)
Cefotaxime	70 (100.0)	0 (0.0)	0 (0.0)
Imipenem	70 (100.0)	0 (0.0)	0 (0.0)
Tetracycline	70 (100.0)	0 (0.0)	0 (0.0)
Trimethoprim-sulfamethoxazole	70 (100.0)	0 (0.0)	0 (0.0)
Meropenem	69 (98.6)	1 (1.43)	0 (0.0)
Piperacillin-tazobactam	63 (90.0)	1 (1.43)	6 (8.6)
Amikacin	63 (90.0)	3 (4.3)	4 (5.7)
Ciprofloxacin	57 (81.4)	2 (2.9)	11 (15.7)
Cefepime	56 (80.0)	0 (0.0)	14 (20.0)
Gentamycin	56 (80.0)	4 (5.7)	10 (14.3)

#### Biofilm formation

Among 70 MDR *K. oxytoca* from stool samples, none of them produced strong-level biofilms and all were in moderate level (Additional file 3: Fig. S3).

#### Antibacterial effects

The mean MIC_90_ and MBC_90_ of curcumin were 250 mg/mL and > 250 mg/mL, respectively. Meanwhile, those of silybin included 500 mg/mL and > 500 mg/mL, respectively. All concentrations of both plants had antibacterial effects against MDR *K. oxytoca* isolates.

#### Gene expression

The calculation of expression levels was performed according to the 2^−ΔΔCT^. We observed that 100 mg/mL of curcumin and silybin could singly decrease the expression of *fimA* and *mrkA* genes, respectively (data not shown). None of other genes were significantly affected. The anti-virulent effect of 100 mg/mL of silybin and curcumin in combination was shown by decrease in the expression of *fimA* (2.1-fold, *P* < 0.0001) and *mrkA* (2.1 fold, *P* < 0.0001) genes (Additional file 4: Fig. S4). Moreover, these compounds decreased the expression of *bla*_IMP1_ (3.2-fold, *P* < 0.0001) gene. Notably, there was no significant effect on expression of *pilQ*, *matB* and *bla*_OXA-48-like_ genes (Additional file 4: Fig. S4).

## Discussion

In this study, 70 MDR *K. oxytoca* isolates collected from stool samples during 2012–2019 were included. All isolates were resistant to ampicillin, ceftazidime, trimethoprim-sulfamethoxazole, imipenem and tetracycline. All isolates carried the *bla*_IMP_, *bla*_OXA-48-like_, *mrkA*, *pilQ*, *matB* and *fimA* genes. We observed that all of them were moderate biofilm producers possibly mediated by adhesins including *mrkA*, *pilQ*, *matB* and *fimA* genes. In line with the current study, Ghasemian et al. [5] from Iran reported high rate of adhesins among MDR and non-MDR *K. oxytoca* isolates during 2016–2017.

There is scarcity in data regarding effects of curcumin and silybin against expression of virulence and antibiotic resistance genes among *K. oxytoca*. Moreover, antimicrobial and anti-biofilm effects of curcumin and silymarin has been exhibited previously [15, 16, 27]. In this study, the mean MIC_90_ and MBC_90_ of curcumin were 250 mg/mL and > 250 mg/mL, respectively. Meanwhile, those of silybin were 500 mg/mL and > 500 mg/mL, respectively. In previous study by Adamczak et al. [15], *Acinetobacter lwoffii* (250 µg/mL), *Streptococcus pyogenes* (31.25 µg/mL), *Pseudomonas aeruginosa* and *Enterococcus faecalis* (62.5 g/mL), and methicillin-sensitive *Staphylococcus aureus* (250 µg/mL) were found to be susceptible to curcumin. In another study by Evren et al. [27], MIC and MBC values were between 60 and > 241 μg/mL and greater than 241 μg/mL, respectively. The inhibition of bacteria may be due to this fact that compounds derived from *S. marianum* and *C. longa* are known to exert profound antibacterial effects, mostly by inhibiting RNA and protein production, quorum sensing (QS) system, and targeting the cellular components [28, 29].

In this study, 100 mg/mL of curcumin and silybin could singly decrease the expression of *fimA* and *mrkA* genes, respectively. None of other genes were significantly affected. The anti-virulent effect of 100 mg/mL of silybin and curcumin in combination was shown by 2.1 fold reduction (*P* < 0.0001) in the expression of *fimA* and *mrkA* genes of MDR *K. oxytoca* compared to control strain. Moreover, these compounds reduced the expression of *bla*_IMP1_ gene. Notably, there was no significant effect on *pilQ*, *matB* and *bla*_OXA-48-like_ genes. In an experiment by Eslami et al. [28], silymarin had no effect on *bla*_IMP_ and *bla*_OXA-48_ expression in MDR *E. coli*; however, curcumin down-expressed *bla*_IMP_. In a previous study by Shariati et al. [30], synthesized nano-curcumins exhibited significant (*P* < 0.001) downregulation of the transcription of some virulence genes in PAO1 (16-fold) and MDR (13-fold) strains of *P. aeruginosa*, respectively. Another study by Kumbar et al. [31], showed that curcumin diminished the virulence of *Porphyromonas gingivalis* by reducing the expression of virulence factors genes. Also, Shen et al. [32], showed that silibinin, a flavonoid that is isolated from *S. marianum*, reduced the virulence of *Streptococcus suis* serotype 2. The decrease in the expression of virulence genes may be related to the effect of the tested compounds on QS genes, which play an important role in regulating other bacterial factors such as pathogenicity, biofilm production, and secretion systems [33]. In conclusion, this study showed the synergistic effects of curcumin and silybin against some of virulence factors and carbapenemase genes in MDR *K. oxytoca*. The results of this study provided a suitable platform for further investigations, especially in vivo experiments to verify these promising effects.

### Limitations

Major limitations of this study included lack of in vivo experiment, low number of samples and no investigation of curcumin and silybin effects on virulence gene expression of other nosocomial pathogens.

## Supplementary Information

**Additional file 1: Figure S1.** Flow chart of the employed procedures in the present study.

**Additional file 2: Figure S2.** Antibiotic resistance patterns of *Klebsiella oxytoca* isolates.

**Additional file 3: Figure S3.** Microtiter plate assay showing moderate level of biofilm formation in different *Klebsiella oxytoca* isolates.

**Additional file 4: Figure S4.** The expression of virulence and antibiotic resistance genes in exposure to 100 mg/mL of combined silybin and curcumin; "c" stands for control group without exposure neither to curcumin nor the silybin, "t" stand for treatment and "df" stand for decrease in fold value.

## Data Availability

The data of the current study are available from the corresponding author on reasonable request.

## Abbreviations

CP: Carbapenemase-producing
MBC: Minimum bactericidal concentration
MDR: Multidrug-resistant
MIC: Minimum inhibitory concentration
MHB: Mueller Hinton broth
