# Supplementary figures and images for: Synergistic effects of silybin and curcumin on virulence and carbapenemase genes expression in multidrug resistant Klebsiella oxytoca
Source: BMC Res Notes. 2022 Oct 22;15:330. doi: 10.1186/s13104-022-06172-3 (PMC9588228; doi:10.1186/s13104-022-06172-3)

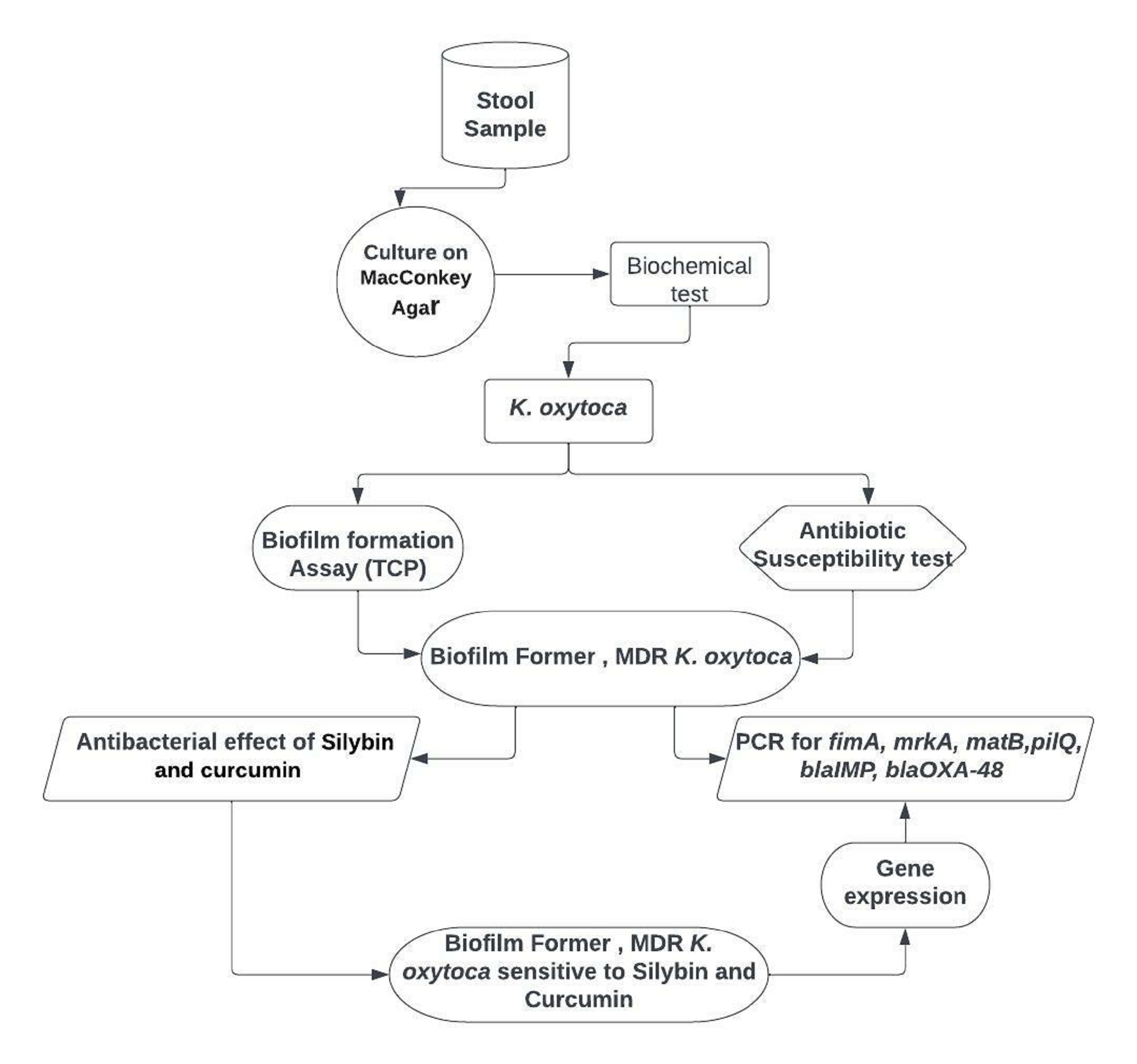

Supplement: Supplementary file 1 — Additional file 1: Figure S1. Flow chart of the employed procedures in the present study. [file 13104_2022_6172_MOESM1_ESM.jpg]

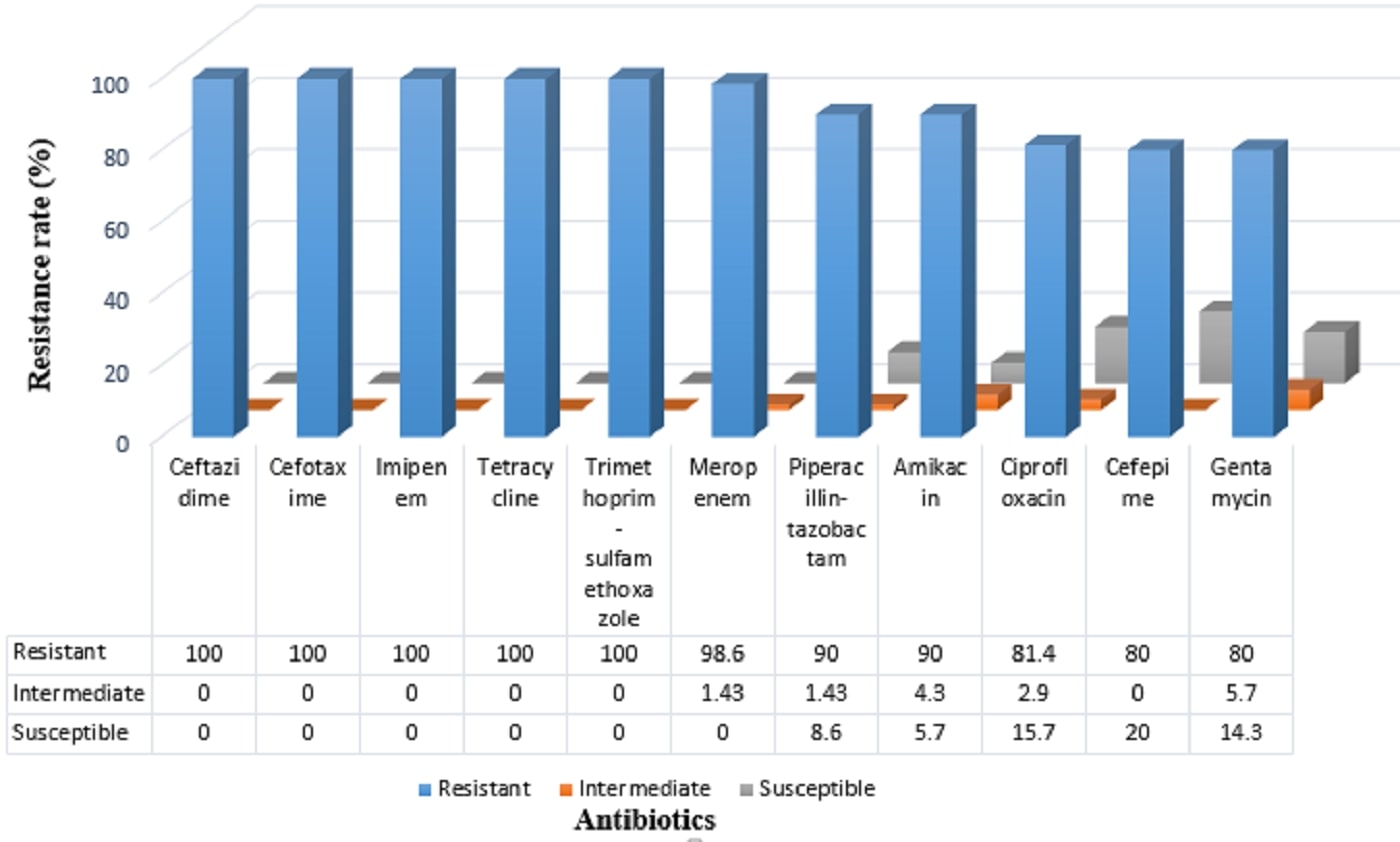

Supplement: Supplementary file 2 — Additional file 2: Figure S2. Antibiotic resistance patterns of Klebsiella oxytoca isolates. [file 13104_2022_6172_MOESM2_ESM.jpg]

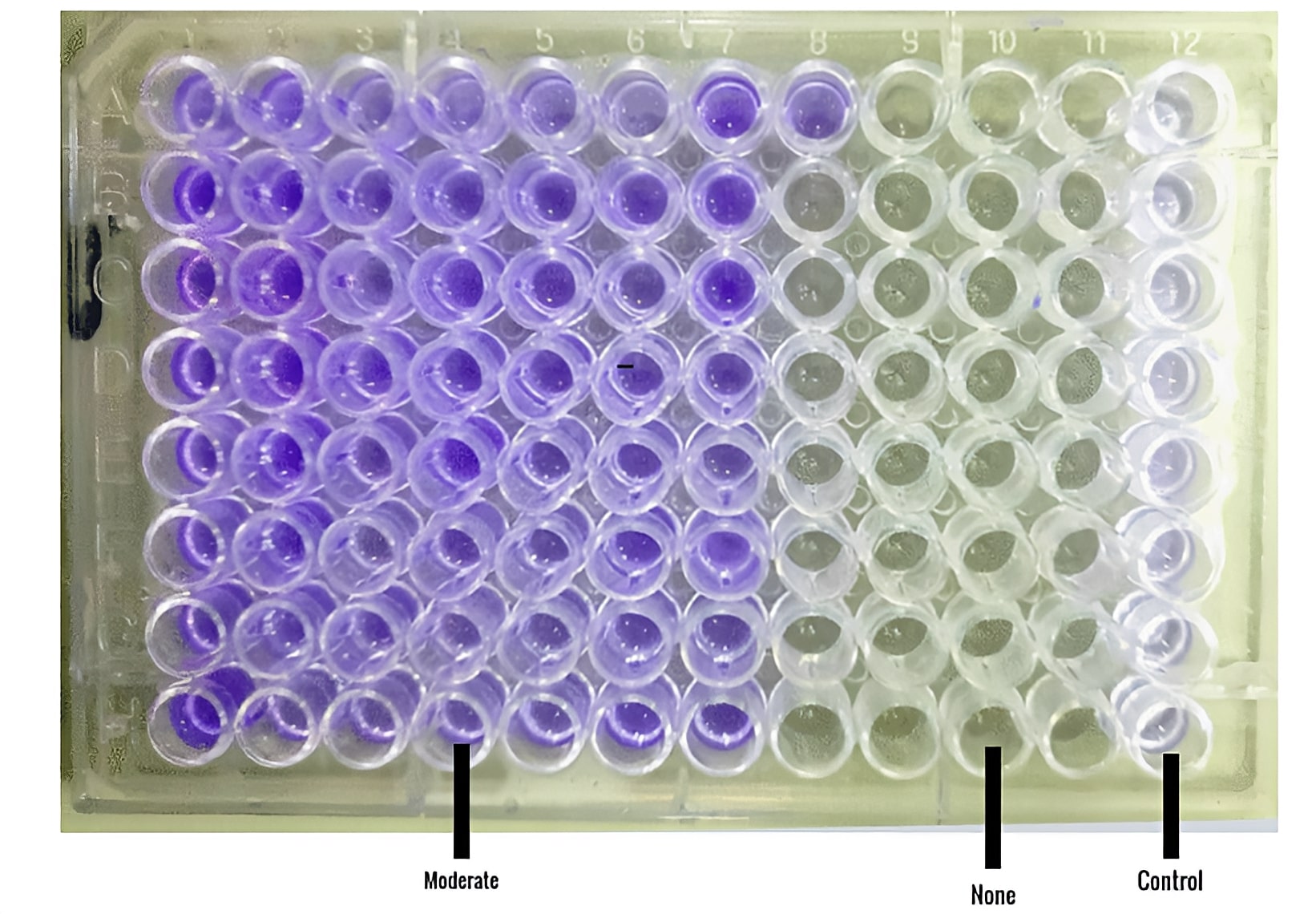

Supplement: Supplementary file 3 — Additional file 3: Figure S3. Microtiter plate assay showing moderate level of biofilm formation in different Klebsiella oxytoca isolates. [file 13104_2022_6172_MOESM3_ESM.jpg]

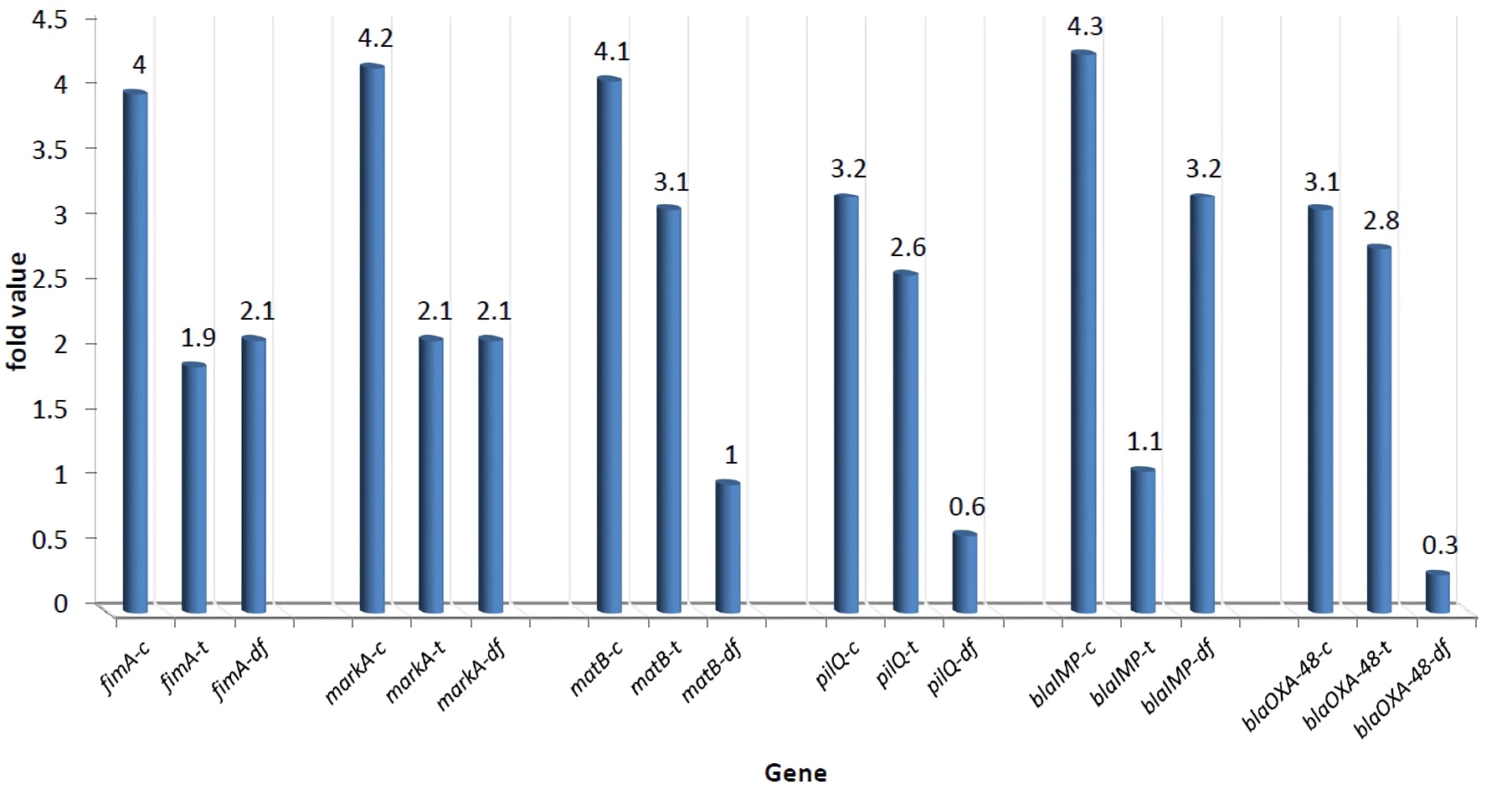

Supplement: Supplementary file 4 — Additional file 4: Figure S4. The expression of virulence and antibiotic resistance genes in exposure to 100 mg/mL of combined silybin and curcumin; "c" stands for control group without exposure neither to curcumin nor the silybin, "t" stand for treatment and "df" stand for decrease in fold value. [file 13104_2022_6172_MOESM4_ESM.jpg]
